# Assessing Risk Thresholds in Controlled Human Infection Models (CHIM)

**DOI:** 10.1111/bioe.70072

**Published:** 2026-03-24

**Authors:** Alexa Nord‐Bronzyk, Barnaby Young, Jerry Menikoff, Julian Savulescu, G. Owen Schaefer

**Affiliations:** ^1^ Centre for Biomedical Ethics, Yong Loo Lin School of Medicine National University of Singapore Singpaore Singapore; ^2^ National Centre for Infectious Diseases; Department of Infectious Diseases, Tan Tock Seng Hospital; Lee Kong Chian School of Medicine Nanyang Technological University Singapore Singapore; ^3^ Uehiro Oxford Institute University of Oxford Oxford UK

**Keywords:** challenge trials, research ethics, risk thresholds, risk‐benefit assessments

## Abstract

Controlled Human Infection Models (CHIMs) are a type of clinical trial involving deliberately exposing human volunteers to an infectious agent. Compared to studies of natural infection, CHIMs offers distinctive benefits, from the ability to study presymptomatic infection to a direct assessment of the efficacy of vaccines and therapeutics in a shorter time and involving fewer participants. Although the CHIMs do not fundamentally differ from other early‐phase clinical trials, they raise a unique set of ethical considerations. Modern CHIMs have been limited to studies which pose very low risk of participants experiencing serious adverse events, and CHIMs which lie on the upper end of risk remain controversial. Setting risk thresholds for more hazardous trials has become a topic of debate leading to various attempts to develop a model to ethically evaluate these risks. While there are now well‐accepted practices around established pathogens, future pandemics may involve more extreme threats to public health and CHIMs may have an important emergency rescue function, with some groups willing to undertake significant risks. We must ask, what risk of death and serious morbidity should future CHIMs permit in the face of such challenges? This paper proposes a taxonomy laying out three possible approaches for thinking about risk thresholds in CHIM: (1) consistency; (2) engaging community perspectives; and (3) no risk threshold. We explore the strengths and weaknesses of each approach and offer a resource for those in the research community to inform their evaluation of the ethical permissibility of current and future CHIM.

## 1 Introduction

Controlled Human Infection Models (CHIMs), otherwise known as challenge trials, are a type of clinical trial that involves deliberately exposing human volunteers to an infectious agent. By directly engineering the exposure of interest, CHIMs aim to supplement traditional research study designs that rely on natural infections, by allowing for immediate, controlled, and precise observation of infection and the monitoring of disease mechanisms, pathogenesis, and participant immune responses [1]. Beginning with physician Edward Jenner's testing of the original smallpox vaccine in 1796, CHIMs have been used for decades to advance our understanding of infectious diseases such as malaria, influenza, typhoid, and cholera. The rise of the COVID‐19 pandemic has since triggered new challenge trial exploration, pushing researchers to consider higher‐risk trials [2].

CHIMs do not fundamentally differ from other early‐phase clinical trials (i.e., Phase 1 studies in healthy volunteers of an experimental drug), yet often raise a unique set of ethical considerations regarding their risk–benefit profile, especially considering social value, evaluation of fair participant selection and compensation, suitable site selection, stakeholder engagement, informed consent, minimal participant benefit, potential for exploitation, and unknown long‐term risks [1, 3]. While there is general agreement that many CHIMs meet the Belmont Report standard that “risks to subjects be outweighed by […] anticipated benefit to society,” the risk profile of each CHIM should nevertheless be evaluated [4].

A recent review by Adams‐Phipps et al. of 276 challenge trials from 1980 to 2021 involving more than 15,000 participants found only 24 serious adverse events (SAEs) reported (< 0.2% of participants) and 0 reported deaths or cases of permanent damage.^1^ [5] Still, the debate continues about the inherent riskiness of intentional exposure and uncertain consequences (e.g., long‐term effects), and CHIMs which lie on the upper end of risk thresholds remain especially controversial.

Rather than evaluate risk of all previously conducted trials, this paper intends to address ways of dealing with these future riskier trials where permissibility hinges on factors such as the availability of effective treatments, severity of disease, and risks to bystanders, as well as risk mitigation strategies such as developing attenuated challenge strains, restricting participation to young healthy volunteers, ensuring the availability of “rescue therapy” where possible, and enforcing quarantine in secure facilities to prevent third‐party infection [3, 6].

This is especially relevant as the field has moved into more complex risk areas in recent years, such as challenge studies with novel pathogens, expanding participation to older adults, and utilizing agents that can cause a chronic infection [7, 8]. This has led to various attempts to develop a mechanism or framework to ethically evaluate risks in CHIMs including quantitative modelling, qualitative methods, decision‐making analysis, and setting risk thresholds, among others [9, 10, 11, 12, 13, 14, 15, 16, 17, 18].

In particular, setting risk thresholds for these riskier trials has become a topic of debate. While some argue these thresholds remain largely underexplored, there are indeed varying strong claims in the literature on this topic [6, 19]. Some argue for a hard cap on risk [16, 20] while others agree there shouldn't be any strict limit on risk and rather that risks should always be justified by the benefits [16, 21, 22, 23]. Others argue that public trust may be eroded if research isn't restricted to no more than minimal risk, and some argue that minimal risk may be too restrictive and ineffective in protecting public trust [22, 24]. This is also complicated by those who may voluntarily participate in a study for altruistic purposes, especially when the risks are significantly elevated [25].

This paper proposes a taxonomy laying out three possible approaches for thinking about risk thresholds in CHIMs: (1) consistency—benchmarks CHIMs risk against risk levels in other activities we generally permit; (2) engaging community perspectives—focuses on engaging the public and incorporating views of those affected, the experts, and other stakeholders; and (3) no risk threshold—where there may be no cap to the level of permissible risks so long as they are justified by the benefits.^2^ Each approach has strengths and weaknesses. The consistency approach is intuitive but faces significant challenges in achieving comparison validity; engaging community perspectives has the advantage of not relying on the particular or idiosyncratic values and judgements of researchers or oversight bodies, but may not always be feasible due to resource constraints; no risk thresholds upholds autonomy and allows for limitlessness in expected benefits, but may fail to adequately protect participants from increased health risks.

While there are now well‐accepted practices around established pathogens, future pandemics such as those involving Avian influenza or new novel coronavirus may involve more extreme threats to public health and CHIMs may have an important emergency rescue function. Similarly, for viruses with high disease burdens such as dengue, we must ask, what risk of death should future CHIMs permit in the face of such challenges?

We note that our points may extend to all non‐therapeutic research, but it is clearest in CHIM because these may often have no therapeutic benefit and involve risk. In the case of therapeutic research, the potential benefits to the individual may justify higher risks, with the exception of Phase 1 trials which are more similar to CHIM. Therefore, variables such as urgency, uncertainty, and social factors may contribute to the need for more flexibility in standard frameworks. While some regulations and guidelines may allow for a level of flexibility, there may be a lack of clarity on how to operationalize this.

Our aim here is not to establish which approach is most suitable for CHIMs, but rather provide an outline of approaches that can inform relevant parties such as investigators, regulators, or oversight bodies that may be considering implementing (or abolishing) absolute risk thresholds. Any such attempts should be transparent in terms of their normative foundations and argumentative basis, so that they can be properly evaluated as well as explicable to relevant parties (such as participants or the public) who will be affected by them. Furthermore, each approach is not necessarily, mutually exclusive. For example, one might employ the consistency approach using mortality rates of other permissible activities as a benchmark resulting in a set risk threshold. Others may compare to different permissible activities where we do not set a risk threshold and thus endorse very significant risks justified by benefits or promotion of autonomy. So elements of more than one approach, or possibly all three, could be employed in practice. The following will outline each approach in an effort to inform CHIMs risk threshold assessment in what has been an ongoing controversial space.

## 2 Approaches to Evaluating Risk Thresholds in CHIM

This section outlines the three approaches mentioned above to evaluating risk in CHIM: (1) consistency; (2) engaging community perspectives; and (3) no risk threshold. Each approach also includes several variants that differ from the overall approach and may be competing with one another. Each approach will be explained and critically evaluated to produce a taxonomy lending to the biomedical research communities' efforts to understand risk thresholds appropriate to the CHIM at hand.

### 2.1 Consistency Approach

The consistency approach benchmarks CHIMs risk against risk levels in other everyday activities we generally permit (i.e., walking, cooking, blood testing), where the permissibility of such activities is also predicated on proper informed consent and aligned with other research ethics guidelines. Consistency with levels of risk associated with previously accepted CHIM can also be applied as a comparator.

Structurally, the consistency approach parallels the minimal risk standard outlined in various biomedical research ethics regulations and literature such as The Belmont Report, Council for International Organizations of Medical Sciences (CIOMS) Guidelines, and Human Biomedical Research Act (Singapore). Each document offers a different version of defining minimal risk along the lines of “an anticipated level of harm and discomfort that is no greater than that ordinarily encountered in daily life, or during the performance of routine educational, physical, or psychological tasks” (p. 9) [26]. Depending on the jurisdiction, minimal risk classification is used for purposes such as determination for eligibility for expedited/exempt review, waivers of consent, or assessment of acceptability of research with minors. According to recent estimates, the mortality risk of an ordinary car ride poses up to a 1 in 300,000, which can be thought of as one benchmark for a minimal risk threshold [27]. The present discussion is on maximum risk thresholds for CHIMs that will likely involve full informed consent and full board ethics committee review, and where there is an intervention with a possible therapeutic effect. Therefore, the minimal risk threshold would be overly restrictive and not applicable.^3^ Nevertheless, the analogical method of establishing minimal risk (identify an acceptable set of risks in one domain and set thresholds for risk in research at a similar bar) can also be applied to maximum risks for CHIMs. Under this approach, CHIMs can be evaluated by comparison to other permissible activities that may carry higher risk (e.g., live organ donation, high‐altitude hiking, cycling, and novel vaccination), including risk levels involved in CHIM conducted thus far.

Comparing these permissible risks with CHIMs risk is often done by looking at the morbidity and mortality rates of each which can result in hard caps on risk, no caps, or other qualitative assessment models. While we do not aim to resolve debates over appropriate comparators, we acknowledge that the strength of the consistency approach depends on the validity of the comparator, with organ donation offering a more fitting analogy than leisure activities due to its clearer third‐party benefit.

#### 2.1.1 Mortality and Morbidity Risk as Comparators

While there is no universally agreed‐upon numerical threshold for what counts as a “greater than minimal” likelihood of mortality and morbidity, there have been various attempts to estimate this number by comparing CHIM risk to risks we accept in daily life. For example, Rid and Wendler, “if the cumulative net risks in the study clearly exceed the general limits of acceptable research risk, the study should be rejected” [28]. But what is this general limit?

If we take the < 0.2% reported SAEs (with zero resulting in death or permanent damage) in the Adams‐Phipps et al. review to suggest there is relatively little controversy among people engaged in these debates that the approved trials are within an acceptable range of risk, then anything above 0.2% might be thought of as a “greater risk” trial, but how far above that are we willing to go?^4^ The review is not a comparator in itself, but reflects the “low risk” level of accepted trials where this level of risk serves as a comparator. While we are not trying to re‐open the analysis of all prior trials, we intend to explore how the consistency approach can, has, and should be employed.

David Resnik offers one approach by comparing permissible risk levels we see in daily life across medical procedures, activities, and occupations. He rather opaquely derives a “1 percent standard” whereby he suggests “study participants should not be subject to greater than 1% chance of serious harm, such as death or permanent disability”(8) [16]. This roughly tracks the lifetime odds of dying from a car crash in the United States which is 1 in 95, whereas the daily risk becomes as low as 1 in 300,000 as mentioned above [27, 29]. In 2019, the yearly risk of dying in a road traffic accident in the United Kingdom was estimated to be roughly 1 in 40,000 [30]. This then raises the question of whether we should be thinking of these risks across a day, a year, or a lifetime.

Various research ethics frameworks (e.g., CIOMS, the Belmont Report) use “daily life” risks experienced repeatedly over time as comparators. However, participation in CHIM is usually a one‐time event which suggest that lifetime risks may serve as more appropriate comparators (e.g., lifetime risk of dying in a car accident). However, “middle‐ground” approaches may also be appropriate. An example might be focusing on the period of elevated risk during the actual exposure and accounting for possible subsequent quarantine/monitoring. This might allow IRBs to assess risk that more closely tracks when harm is most plausibly incurred and avoids inflation of long‐term exposure. Minimal risk regulations usually stipulate “daily life” risks as comparators, but since participation in a riskier challenge trial is likely a one‐time event across someone's life, it may be more appropriate to consider lifetime risks.

Live organ donation has frequently been touted as an acceptable comparator with a mortality risk of 0.03% to 0.5% [3, 31, 32]. In the early days of the COVID‐19 pandemic, some argued that SARS‐CoV‐2 CHIMs with estimated mortality risks of 0.03% and hospitalization risks around 1.1% were too risky [3]. However, these estimates track the epidemiological data from estimates of the severity of the coronavirus disease [33] rather than the mortality risk posed by CHIM with the appropriate risk mitigation strategies in place (i.e., refining eligibility criteria, close monitoring, etc.), which then the authors admit would make the risks negligible.

These overestimates may arise because estimating mortality risk in CHIM is usually measured by looking at the Infection Fatality Ratio (IFR) for populations mirroring the participant population. This was the method the charity 1 Day Sooner used in 2021 to find the estimated mortality risk for healthy COVID‐19 CHIM participants to be approximately 1 in 10,000—“half the mortality risk of liposuction, a third of living kidney donation, and 500 times lower than venturing into space as an astronaut” [10, 25]. The IFR of a disease is the percentage of people infected with the disease that are expected to die, but any major CHIM will have various safeguards in place reducing these risks. Thus, as Eyal and Wendler point out, SARS‐CoV‐2 is now far less lethal per infection placing current mortality risk estimates at 0.0001857%, or 1 in 537,634 (2025), making a SARS‐CoV‐2 CHIM, they argue, fit with the minimal risk definitions of various global regulations including the CIOMS/WHO guidelines [27].

Others have compared acceptable risk in CHIM to other permissible activities that are sometimes done at least partly for altruistic motivations such as firefighters and military personnel [20, 34] with mortality risks of 3.4 deaths per 100,000 (0.0034%) fire incidents and 37.6 deaths per 100,000 person‐years, respectively [16, 35]. Altruistic and voluntary pursuits, argue Jayaram, Sparks, and Callies, make for good comparators as challenge trial participants may often be voluntarily taking on risk for altruistic reasons which is what would make live organ donation a suitable comparator and skydiving less so [31].

But what about the threshold for CHIM involving pathogens which can cause a chronic illness or for which there is no highly effective treatment available as risk mitigation (e.g., hepatitis B, schistosomiasis, dengue)? These inevitably raise additional concerns, but the social impact may also motivate a greater risk threshold [36]. Some argue for the principle of risk parity to vaccine research—“if it is permissible to expose some members of society (e.g., health workers or the economically vulnerable) to a certain level of ex ante risk in order to minimize overall harm from the virus, then it is permissible to expose fully informed volunteers to a comparable level of risk in the context of promising research into the virus” [37]. Should these trials be compared to the riskiest permissible daily activities (alcohol consumption, unprotected sex, smoking)? Some might argue these activities aren't ethically permissible, but they are certainly legal and it is often considered an impingement on autonomy if regulations prohibit individuals from participating in them. But, as Resnik rightly points out, socially acceptable doesn't mean ethical [16]. The ethical considerations involved in these comparisons might include measuring other variables such as harm to others (where harm to others applies), thus making mortality and morbidity controversial comparators.

#### 2.1.2 Countervailing Considerations

The consistency approach thus faces significant challenges in achieving comparison validity—for any given comparison activity, there will likely be morally relevant disanalogies. Firefighting, for instance, involves a clear and pressing danger with identifiable individuals' lives at stake, while the benefits gained from CHIMS are more uncertain and causally removed. As noted by Rid, employing such comparators also faces challenges in determining when and if non‐research risk might be ethically permissible as well as sufficiently analogous to the risks in research [38]. Furthermore, while we may have empirical data stratifying the risks of routine activities, the risks associated with certain CHIM may be more uncertain. This was the case during the COVID‐19 pandemic when discussion around CHIM was in hard debate, but the uncertainties were associated with a rapidly evolving novel pathogen (i.e., long covid) [3, 39]. Therefore, some may critique the consistency approach for failing to account for epistemic uncertainty inherent in CHIM.

Another challenge for the consistency approach is that it presumes the moral permissiveness of comparator cases. Some socially sanctioned activities may nevertheless be ethically objectionable, such as exploitative contracts or unsafe working environments. A successful argument by analogy may need to establish not just that an analogous activity is permitted, but that there is good ethical reason to permit it. This is why the analogies we are generally accepted to be permissible activities. The flip side of this challenge is that there may be some activities we should permit, but due to excessive protectionism, those activities are prohibited (as, e.g., some have argued people should under certain circumstances be permitted to donate both their kidneys) [40]; as a result, undue conservatism in other domains could also spread to CHIM under a consistency approach.

### 2.2 Engaging With Community Responses

The second approach in this paper focuses on engaging with community responses. While community engagement is typically a complementary part of ethical review, we propose that –without limiting its broader roles—it could help resolve the specific policy‐level question of appropriate risk thresholds in CHIM studies, though we acknowledge and address the limitations of relying solely on public opinion.

The Centers for Disease Control and Prevention (CDC) defines community engagement as “the process of working collaboratively with and through groups of people affiliated by geographic proximity, special interest, or similar situations to address issues affecting the well‐being of those people” [41]. This approach aims to ensure local relevance of the study, enhance transparency, and trust with the local community, as well as address power inequities. Engaging the public is widely endorsed in the literature and in policy, such as in the United States under the Exception From Informed Consent (EFIC) regulatory stipulation and is also often a prerequisite to many CHIM in formats including surveys, discussion groups, and workshops [14, 42, 43, 44]. Engaging with the community has been described using various terms such as Patient and Public Involvement (PPI), Community‐Based Participatory Research (CBPR), and Participatory Action Research (PAR). Community and Public Engagement (CPE), for example, is endorsed by the United Kingdom. The National Institute for Health Research (NIHR) with robust CPE requirements for funding, monitoring, and evaluation of health research [45].

In the context of risk thresholds, a community engagement approach would push the contentious question to affected communities. Whether a given CHIM is excessively risky would then be dependent on whether the community involved finds that risk level acceptable. This process operates separately from consent, insofar as even if some individual members of a community may be willing to accept a given risk level, if the broader community finds the risks too high that the study cannot go forward.

Community engagement has the advantage of not relying on the particular or idiosyncratic values and judgements of researchers or oversight bodies to determine acceptable risk thresholds. Instead, the question is devolved to the community. At the same time, because it does not rely wholly on individual consent, the approach retains the character of putting some reasonable limits on research activities even when particular participants are agreeable. The question then turns to how heavily community engagement should be weighed.

We cannot ignore public preferences as even the most robust philosophical theories are susceptible to biases and framing effects too [46]. The public is of course also subject to the same criticism, but we cannot ignore public preferences because trials may become non‐viable if they lack a social licence due to being excessively risk, provoking backlash and loss of trust and legitimacy. Nonetheless, if a certain community deems a CHIM to be unethical, should that outweigh even the most potentially beneficial trial? How should the research community handle the public's opinions fairly where biases and political motivations may be at play?

#### 2.2.1 Meaningful Engagement

Some have dealt with these questions through promoting deliberative democracy which engages diverse stakeholders in structured discussions to develop ethical frameworks for policy and practice [47, 48, 49]. The focus is on consensus‐building, inclusive participation, justification, and dynamic and revisable decision‐making [50, 51, 52].

The Oregon Health Plan is one example of deliberative democracy, which invited Oregonians to provide their insights regarding which medical conditions should be covered by the state's Medicaid program [53]. More recently, Canada's NICE's Citizens Council has passed legislation mandating public deliberation structures advising the pharmaceutical benefits program and performance of the Quebec health system [54]. Shah, Miller, and Lynch also illustrate the importance of community engagement for research risk to bystanders through a Zika CHIM by employing consultation methods such as incorporating local knowledge about behaviours, risks, and concerns regarding the trial [55].

Focusing on community engagement is also considered especially important when evaluating CHIM in low‐ and middle‐income countries (LMIC) where research agendas may not always reflect the local perspectives and needs. Also, many pathogens are endemic in LMIC making these countries better study sights because those participants are those most affected. Additionally, risk may be lowered with participants who may have previously built immunity [56].

#### 2.2.2 Collective Reflective Equilibrium in Practice (CREP)

One notable approach to community engagement is the Collective Reflective Equilibrium in Practice (CREP) approach developed by Julian Savulescu, Christopher Gyngell, and Guy Kahane. CREP is described as “a deliberative process that looks for coherence between attitudes, behaviours and competing ethical principles” [57].

According to CREP, public attitudes may help to inform policy, but not without proper evaluation. The CREP method thus seeks to develop policy as follows:

1. Gather the preferences and intuitions of the public as well as experts through surveys, focus groups, citizens juries, expert body statements, and relevant academic literature.
2. Screen the gathered data from the first step by scrutinizing the intuitions for bias and prejudice, giving weight to convergence between the public and expert intuitions, and giving weight to revealed overstated preferences.
3. Seek overlapping consensus by assessing consistency between laundered preferences and major ethical theories/principles, and giving weight to preferences when normative frameworks conflict, while also applying ethical theories, mid‐level principles, guidelines, and declarations.

Grimwade et al. applied CREP by performing a cross‐sectional study in the form of two online surveys—one for CHIM experts and one for UK citizens—developed in collaboration with experts in experimental psychology, CHIM and bioethics, and pretested by a group of medical students [58]. Survey participants were asked how strongly they agree or disagree with CHIM participants being allowed to participate in various hypothetical CHIM on a scale of 0: strongly disagree to 6: strongly agree. The public's attitudes towards risk of death were more risk‐averse compared to those of professionals—“across all levels of risk and over the two risk categories, experts' willingness to allow participation (WTAP) was higher than public WTAP (*b* = 0.32, *p* < 0.001) and experts' risk threshold with payment was lower than public payment (*b* = −0.41, *p* < 0.001)” (p817) [58]. Insights like these can be very useful rather than relying only on the intuitions of investigators. The public's willingness to pay for higher risks might suggest an implicit threshold allowing for higher payments for greater risks, but there is not a clear cap on how much risk the public believes is ethically justifiable.

#### 2.2.3 Countervailing Considerations

Feasibility of engaging the community and other consultative empirical bioethics research methods may be a challenge when urgency is paramount and resources are slim. Gathering information and adequately informing each party takes significant planning and resources which may not always be achievable. Complications like these have led some to argue that community engagement may or may not be necessary, especially in the case of urgent vaccine studies during a pandemic, and may even be *more* necessary prior to field trials than prior to CHIM [59]. Researchers should be careful to balance respecting public perceptions and preferences with those of the experts, ensuring these data do not hinder critical advancements or reflect misinformation.

### 2.3 No Risk Threshold

The final approach in this paper describes those who do not endorse any strict risk thresholds. These authors argue that CHIMs with higher risk of morbidity and mortality than what is usually acceptable can be ethically justifiable where benefits such as social value, the possibility of high‐quality of informed consent, and lives saved are great enough to warrant such risks [14, 15].

Importantly, this is also the ethical and regulatory standard globally for approving CHIM [16]. An outlier lies in the Nuremberg Code which states, “No experiment should be conducted where there is an a priori reason to believe that death or disabling injury will occur; except, perhaps, in those experiments where the experimental physicians also serve as subjects” [17]. Additionally, the CIOMS 2016 states “some risks cannot be justified, even when the research has great social and scientific value and adults who are capable of giving informed consent would give their voluntary, informed consent to participate in the study” [60]. The WHO also states studies posing “serious or irreversible harm” should not be conducted irrespective of potential benefits (p. 12) [61].

While other “no risk threshold” arguments have been laid out above, the focus of this section will be on (1) holistic assessments of risk that take in an array of variables when evaluating risks and benefits, (2) algorithmic and modelling approaches that evaluate a distinct set of variables to evaluate risk, and (3) approaches upholding autonomy.

#### 2.3.1 Holistic Assessments of Risk

A holistic assessment of risk considers various factors including public attitudes, governance structures, and robustness of consent. Because holism admits of a variety of factors that weigh up against each other, it would not generally admit of hard‐and‐fast absolute maximums—acceptable risks will vary by context. Note also that holistic assessments are not essential to the no‐threshold category, but present one variant.

One approach from Williams et al. aims to establish “a determinate decision‐procedure for when to conduct and not conduct CHIMs before and during pandemics” (2). The framework consists of two key questions: (1) Is the proposed study in the public interest? (2) Does the proposed study satisfy other ethical requirements? (2). Justification includes a scientific rationale, a justifiable risk‐benefit ratio, external review, and fulfilment of other ethical requirements including fair compensation, voluntary and sufficient informed consent, appropriate recruitment, protection of the local community, and comprehensive stakeholder communication plans.

The authors argue that higher‐risk studies can be justified only when they offer proportionately greater public benefits, meaning that such studies must meet a much higher benefit‐risk ratio than lower‐risk ones (p.25). They offer the following multiplier approach to calculate a justifiable benefit‐risk ratio:

the ratio of expected population benefit to expected participant harm only needs to be 1:1 in low‐risk studies, needs to be 100:1 in medium‐risk studies, for high‐risk studies should be 1000*x2:1 where x is the ratio of risk to 1% (or 1000:1 when the risk to participants is considered high but below 1%). For example, this would result in a study with a 10% mortality risk requiring a benefit to harm ratio of 100,000:1.

(p. 26)

They admit this model is in place to motivate further discussion for a more optimal approach and stress the need for flexibility by accounting for other factors such as “population or participant enthusiasm for the study or the degree of uncertainty in estimates of benefit and harm” (p. 26).

Despite the 2021 World Health Organization's cap on risk mentioned above, their recent draft guidance for key criteria for ethical acceptability of CHIM during emergencies offers a holistic ethical assessment strategy with criteria including scientific justification, benefit‐risk assessment, co‐ordination, consultation and public engagement, site selection, participant selection, expert review, and informed consent [14]. The draft emphasizes evaluating the social value of a CHIMs while recognizing the complexity during public health emergencies. While the no risk threshold is set, the document suggests potential benefits must outweigh risks for participants, society (in general), and third parties, and consider community infection rates, if the vaccine will provide lasting protection, and the possibility of vaccine‐enhanced disease (pp. 12–13).

#### 2.3.2 Algorithmic and Modelling Approaches

Some have suggested stricter algorithmic and modelling approaches to evaluating risks and benefits using quantitative methods to calculate whether the benefits outweigh the risks. This approach may seem utilitarian insofar as it is limited to consideration only of risks and benefits (so, e.g., community opinion wouldn't impact the risk‐benefit calculation) and in theory any level of risk could be justified when the prospective benefits are high enough. Nevertheless, the approach is compatible with non‐utilitarian frameworks that hold research studies must have a positive risk‐benefit ratio. Arguably, this approach is implied by various regulatory frameworks that require IRBs to ensure proportionality (i.e., that the risks of a study are proportionate to its benefits, without mentioning risk thresholds) [23].

David Manheim et al. explore the risks of a severe acute respiratory syndrome coronavirus‐2 (SARS‐COV‐2) dosing study with an interactive model. By incorporating new data on IFRs to patients, they use a Bayesian evidence synthesis model to infer rates of hospitalization and estimate individual risk, which can then be extrapolated to overall mortality and hospitalization risk in a dosing study [10]. However, they only estimate one half of the proportionality question (risk level), without assessment of commensurate benefits that would be necessary in a full proportionality test.

Bilinski et al. adopt a more robust proportionality approach by developing a computational model assessing the risks and benefits of CHIM for hepatitis C vaccine development [9]. While admitting considerable uncertainty around parameters, they include varying assumptions such as trial duration, number of candidates, and vaccine uptake, as well as benchmarking against the purported financial burden. Their model can help evaluate risk for future vaccine development, but they urge that each CHIM be evaluated with context‐specific considerations.

Dan Brock uses the case of cost‐effective analysis (CEA) for new vaccine development to demonstrate that while quantitative modelling offers certain benefits, it also poses ethical concerns such as the difficulty of embedding fairness, when discount rates might unfairly disadvantage prevention programmes whose benefits take years to manifest, and knowing which non‐health‐related factors should be included in the model [62]. More recent access to big data has also been recognized to “fast track” decisions on risk mitigation, but there is no consensus about what constitutes professional competencies or duties making it difficult to assess whether models are appropriate [63]. It is also reasonable to imagine quantitative modelling risks dehumanizing participants by reducing the complexity of a person's experiences (e.g., pain, fear) to metrics like Quality‐adjusted life‐years (QALYs) or Benefit Risk Ratio (BRRs).

#### 2.3.3 Approaches Upholding Autonomy

One might argue that provided sufficient informed consent, if an adult wishes to participate in a higher‐risk study, they should be able to do so, just as we allow other high‐risk activities. This would seem to align with John Stuart Mill's harm principle—“the only purpose for which power can be rightfully exercised over any member of a civilized community, against his will, is to prevent harm to others. His own good, either physical or moral, is not a sufficient warrant” [64]. Applied to medicine, the harm principle defends patients against paternalistic decisions on the part of doctors, even if the doctor was trying to preserve the wellbeing of the patient.

Nonetheless, it is fairly well understood, in terms of the long‐standing ethics and regulation of research that we do indeed accept that there are limits on autonomy [65, 66]. These limits become especially important when individuals have diminished capacity for self‐determination, as well as in the case of vulnerable populations. The Belmont Report and Declaration of Helsinki thus have protections in place to safeguard these individuals.

Robert Steel recognizes these limitations while adding that it is the participants' values that matter—“even studies with extremely unfavourable risk profiles can still treat participants in an ethically acceptable way, provided that both the goals of the study and the methods of subject selection are sufficiently sensitive to the values of those who enrol”. For Steel, caps on risk cannot be justified by the paternalistic motivation to protect participants from a potentially poor choice to enrol [67].

Additionally, anti‐paternalism might suggest that proportionality tests may be unethical because they paternalistically restrict people from taking on risk. A proportionality test means prohibiting (or at least, requiring amendment to) a study whose risk‐benefit ratio is deemed unacceptable by a relevant oversight body such as an ethics committee. This is a form of overriding or pre‐empting participants' ability to decide for themselves whether a given risk‐benefit ratio is acceptable during the consent process. It is paternalistic to the extent that the motivation for the restriction on participants' autonomy is to protect the participants' interests. Thus, autonomy‐centric libertarian approaches would eschew not only risk thresholds but also proportionality tests that are generally required to justify CHIM risks.

#### 2.3.4 Countervailing Considerations

The UKRI report and WHO guidance offer holistic approaches to risk assessment by evaluating a wide array of variables and incorporating dynamic strategies that are context specific and all‐encompassing. However, the lack of clear boundaries may create challenges for investigators to employ them appropriately. These approaches differ from more utilitarian approaches (such as the algorithmic and modelling approaches to be discussed below) both in the non‐linear way risks are assessed and in the emphasis on stakeholder engagement. Indeed, the latter element highlights how these three taxonomies are not necessarily, mutually exclusive; actual approaches could be hybrid in nature drawing from a mix of two or more means of assessing risk thresholds. For example, how much social value a study generates may be compatible with a threshold on permissible risk as well as having no risk threshold.

For more qualitative, rule‐based, or deontological approaches, one might argue that the lack of clear risk thresholds leaves room to justify nearly any risk, even a substantial risk of death. It is true that, following from the no‐risk threshold approach, in theory a CHIM with a substantial risk of death could be ethically permissible, if the prospective benefits in terms of lives saved (or other outcomes) are great enough. However, defenders of a no‐threshold approach could point out that this is an extreme and unlikely case as CHIMs are not done using pathogens that are likely to cause death, there is proper informed consent, and participants are healthy and screened to avoid complications [56]. Furthermore, a high risk of death would require a great degree of evidence of potential benefit that may be infeasible for CHIMs due to the inherent uncertainties involved in deriving benefits from biomedical research. Thus, in practice, taking a no‐threshold approach would often not lead to CHIMs being approved that have a substantial risk of very serious harm or death.

## 3 Conclusion

Like the setting of a risk threshold, the choice of the framework to establish a risk threshold itself involves choosing a particular normative approach: consistency with other practices, community and stakeholder engagement, or applying a libertarian or proportionality approach, or applying some other purely normative approach such as utilitarianism. We face a regression problem. How do we decide which approach to use?

Each approach to setting risk thresholds offers strengths and weaknesses that may make it more or less valuable and applicable depending on the context of the CHIM (See Table 1 and Annex 1).

**Table 1:** Summary of each approaches' strengths, weaknesses, and illustrative CHIM application.

Approach	Strengths	Weaknesses	Illustrative CHIM application
Consistency Approach	Clear, structured benchmarks using permissible risk levels from daily activities and previous CHIM. Aligns with established biomedical research ethics guidelines. Can reflect societal norms.	Difficulty achieving comparison validity due to different risk perceptions. May fail to account for uncertainties in CHIM risk estimation. Risk thresholds potentially arbitrary or biased.	A CHIM testing a novel influenza strain, using live organ donation and previous CHIMs as benchmarks, ensuring alignment with existing safety data.
Engaging Community Perspectives	Context‐sensitive and aligns with public values. Enhances trust and transparency, reducing risk of controversy. Incorporates diverse perspectives and local knowledge.	Feasibility issues especially in urgent public health emergencies. Resource‐intensive and risks undue influence from dominant or influential groups. Public opinions may be influenced by misinformation or biases.	A malaria CHIM conducted in a LMIC where engaging the local community helps ensure alignment with local values.
No Risk Threshold	Upholds autonomy, allows case‐by‐case assessment, and risks are justified by benefits, ensuring risk proportionality. Avoids arbitrary or rigid risk caps, allows for greater flexibility. Aligns with regulatory principles and concerns for well‐being.	May permit excessive risk exposure due to lack of clear boundaries. Challenges in quantifying and balancing risks and benefits in a consistent manner. May undermine public trust if risks appear unchecked or arbitrary.	A CHIM for a potential pandemic pathogen with high mortality but also high public health benefit, where strict caps on risk could hinder urgent research.

What is of particular importance is that decision‐makers explicitly share their reasons for choosing one framework over another, or when applying more than one framework to a case. Some of us have previously provided arguments supporting the CREP approach but these arguments are not conclusive [57, 68]. Different approaches need to be justified and clear justified thresholds for death and serious morbidity need to be set, related to the particular context and communicated to participants. The arguments in this paper aim to serve as a resource that those involved in these debates can think about setting a risk threshold in particular CHIM (Table 2).

**Table 2:** Summary of distinctions among consent, therapeutic effect, and comparator type.

Distinction	Option 1	Option 2	Option 3
Consent	With consent—Higher risk may be ethically justifiable because participants have fully informed consent, but consent alone is not sufficient (i.e., Declaration of Helsinki, WHO 2021)^a^	Without consent—Stricter risk thresholds may apply but capped at minimal risk (i.e., CIOMS 2016)	N/A
Therapeutic Effect	Therapeutic research may provide clinical benefits to justify risk, with minimization strategies in place	Non‐therapeutic research may justify risk by societal or scientific value. (i.e., CIOMS 2016 Guideline 4)	N/A
Comparator Type	Routine clinical procedures, such as a blood draw	Daily life activities such as driving a car	Altruistic activities such as firefighting

^a^ WHO 2021 Guidance on the Ethical Conduct of Controlled Human Infection Studies admits that “Even with such criteria in place, participants may still face absolute risks or levels of uncertainty related to SARS‐CoV‐2 infection that might be higher than some other ethically acceptable “non‐therapeutic” studies involving risk to healthy volunteers (e.g., some phase I drug trials and many well established challenge studies), although still within acceptable upper limits to research risk”(p.44). (See reference 61).

## Conflicts of Interest

Julian Savulescu is a bioethics committee consultant for Bayer and a bioethics advisor to the Hevolution Foundation. Barnaby Young discloses honoraria/speaker fees from AstraZeneca, Gilead, Moderna, Pfizer, and Sanofi.

## Endnotes

1 It is notable that the 24 reported SAEs is also an overestimate as all SAEs were counted whether they were or were not related to study procedures. The report states “AEs among challenged participants that were not related to challenge (such as AEs related to vaccination or drug treatment) were not extracted,” so SAEs were still reported regardless of whether they were directly caused by the challenge procedures or not (e.g., plasmodium and salmonella) (611, 615). Additionally, the SAEs reported were fairly minor in that they resulted in brief hospitalizations for observations or supportive care, and did not require invasive interventions (614).

2 Różyńska (2015) has proposed another potential category in the context of high‐risk research which could theoretically be applied to CHIMs: discretion of ethics committees or other oversight bodies. We have not included this in our taxonomy, though, because committee judgment is not itself a distinctive category but a higher‐order decision‐making process our taxonomy can inform: committees must themselves make decisions grounded in reason and evidence, so will need to appeal to one of the other three taxonomical categories in any case when making their judgment. Committees might, for instance, use analogical reasoning to set CHIM risk thresholds (approach 1); consult with members of the public, or possibly their own expert judgments (approach 2); or decide that there is no absolute threshold and evaluate risk‐benefit ratios of proposals on a case‐by‐case basis (approach 3). For the same reasons, we would also reject approaches that come from deriving the threshold from first principles or other fundamental considerations (e.g. from the no‐harm principle; or from the formula that no one should be treated as a mere means to others' aims; or from the notion that some sacrifices are too much to even ask‐‐see e.g. work by Alex E. Voorhoeve on non‐aggregation).” (Citation: Różyńska, J. (2015), On the Alleged Right to Participate in High‐Risk Research. Bioethics, 29: 451‐461. https://doi.org/10.1111/bioe.12146)

3 See Table Two for a summary of relevant ethical factors distinguishing between maximal risk thresholds when there is consent/when there isn't; when there is a therapeutic effect to the intervention or the trial/when there is none; a comparator of a certain kind/a different kind/a third kind.

4 For non CHIM studies, mortality risk was found to be even lower. A 2015 systematic recent review of Phase 1 studies (excluding CHIM) by Rebecca A. Johnson and colleagues analysing adverse events in 475 phase I trials involving 27,185 healthy participants found the median rate of SAEs per treatment group to be zero, and over 98% of these trials reported no drug‐related SAEs, with a 0.03% chance of SAEs. (Johnson, Rid, and Wendler 2016).

## Annex 1

### 1.1

We acknowledge one limitation of our paper is that, in exchange for the depth of coverage of individual taxonomical categories, we are not able to fully elaborate on a synthetic approach of taking these taxonomies together or a decision‐making process for adjudicating between them, beyond some broad gestures. The following case study is provided as a resource illustrating how the three approaches may diverge.

**Example case study: A dengue CHIM**

Dengue is a mosquito‐borne disease causing widespread infection globally, and there is a lack of reliable treatment. Dengue can cause severe disease with symptoms including dengue haemorrhagic fever (DHF) and dengue shock syndrome (DSS), and sometimes death. The development of a vaccine could significantly reduce disease burden but also comes with a 0.02% chance of a serious adverse event. There is also no guaranteed rescue therapy.

The consistency approach might employ comparators such as live organ donation risk (0.03%–0.5% mortality) and known low‐risk CHIMs (< 0.2% SAE rate) making it likely that the trial might not be permissible as it lies on the borderline of comparator risk.

The community engagement approach may look to the opinions of the local community, stakeholders, and participants. If there is collective agreement that the disease burden justifs elevated risks, the trial may be permissible.

According to the no risk threshold approach, as long as participants are fully informed, respect for autonomy is upheld, and there is a high benefit‐risk‐ratio, the trial would likely be permissible.
